# Efficacy evaluation of a bivalent subunit vaccine against classical swine fever virus and porcine circovirus type 2

**DOI:** 10.1038/s41598-024-53624-w

**Published:** 2024-02-06

**Authors:** Yu-San Chen, Chang-Ye Lee, Chi-Chien Wu, Pei-Lun Kao, Tai-An Chen, Yahui Huang, Wen-Bin Chung, Tsun‑Yung Kuo, Charles Chen

**Affiliations:** 1 Schweitzer Biotech Company Ltd, Taipei City, Taiwan; 2 Department of Biotechnology and Animal Science, National Ilan University, Yilan County, Taiwan; 3 Research Center for Animal Biologics, National Pingtung University of Science and Technology, Pingtung County, Taiwan; 4 Temple University, Philadelphia, PA 19122 USA

**Keywords:** Vaccines, Recombinant vaccine

## Abstract

Classical swine fever virus (CSFV) and porcine circovirus type 2 (PCV2) are two of the most devastating and economically significant pathogens affecting pig populations worldwide. Administration of a combination of vaccines against swine pathogens has been demonstrated to be as efficacious as the administration of single vaccines. In this study, we developed and tested a novel bivalent subunit vaccine against CSFV and PCV2. The safety and efficacy of this vaccine were demonstrated in mice and specific pathogen-free (SPF) piglets. In addition to investigating the serological responses after immunization, challenge studies with both viruses were also conducted. The results showed that this CSFV/PCV2 bivalent vaccine elicited a high level of neutralizing antibodies against both viruses and provided protection in challenge studies. In conclusion, the CSFV/PCV2 bivalent vaccine is safe and effective against CSFV or PCV2 challenge.

## Introduction

Classical swine fever virus (CSFV) and porcine circovirus 2 (PCV2) are significant pathogens which lead to severe economic losses in the swine industry in many countries^1,2^. CSFV belongs to the genus *Pestivirus* of the family *Flaviviridae*, and is a 12.3 kb virus with plus-stranded RNA^3^. It encodes four structural (C, E^rns^, E1, and E2) and eight non-structural proteins^4^. CSFV is classified as a World Organisation for Animal Health (WOAH) notifiable pathogen and the outbreaks of CSFV infection lead to serious restrictions on the international trade of pig-related products. Although several countries, such as United States, Canada, and New Zealand, have successfully eradicated CSFV infection, there are sporadic CSFV outbreaks in some Member States of the European Union, despite its status-of-freedom for CSF^5^. Even in a country with CSFV-free status, the virus could reemerge, such as the outbreak in Japan in 2018 after an absence of more than a quarter of a century^6^. In endemic CSFV regions, vaccination is still a useful and effective way to control the disease and acts as an assistant tool in eradication programs. However, accumulated evidences showed that the emergence of new CSFV genotypes was caused by systematic inefficient vaccination^7,8^. Since the virus remains endemic in wild boar populations, it is imperative for the scientific community to develop more efficient vaccines against future CSFV outbreaks^5^.

PCV2, which belongs to the genus *Circovirus* of the family *Circoviridae*, is a circular, single-stranded DNA virus with a size of 1.76 kb^9^. PCV2 infection can result in a subclinical infection or a porcine circovirus-associated disease (PCVAD), which may manifest as post-weaning multi-systemic wasting syndrome (PMWS), porcine dermatitis and nephropathy syndrome (PDNS), reproductive failure, respiratory disease, and enteritis^10^. Since the first PCV2 commercial vaccine was introduced in 2006, more and more vaccines against PCV2 have been developed and commercialized to control the disease^11^.

Technologies for subunit vaccine production are widely used in human and veterinary medicine. Eukaryotic expression systems, including baculovirus/insect cell system and mammalian cell expression system, possess several advantages, such as the ability to produce proteins with post-translational modifications, high stability and safety profiles, and high protein yields^12^. Such technologies have also been applied to the development of CSFV and PCV2 vaccines. The E2 gene of CSFV (CSFV E2), which is the major structural and immunogenic glycoprotein, induces protective humoral and cellular immunities in pigs against CSFV infection^13–15^. Several commercial vaccines based on CSFV E2 glycoproteins are available and have been applied to field use^15,16^. Furthermore, commercial PCV2 subunit vaccines based on the capsid protein encoded by open reading frame 2 (ORF2) have been proven to induce protective immunity in field studies^17,18^. PCV2 vaccines based on genotype 2a also revealed cross-protection against PCV2b and PCV2d challenges^19,20^.Vaccination against CSFV is mandatory up to the end of 2022 as part of the CSFV eradication program in Taiwan. The number of annually imported and inspected PCV2 monovalent and PCV2/*Mycoplasma hyopneumoniae* combined vaccines was 8.6 million doses in 2021. A total of 8.1 million pigs were slaughtered in the same year. These facts indicate that almost all pigs produced in Taiwan were vaccinated with CSFV and PCV2 vaccines. Furthermore, the reemergence of CSF in Japan mentioned above has gained increased attention, and regular vaccination with a live attenuated GPE- vaccine was started in October 2019^21^. Co-infection of PCV2 and CFSV has also been reported. The prevalence of co-infection varied among areas/countries, with a marked difference between 13.06% in Shandong province in China and 73.91% in Cuba^22,23^. Both PCV2 and CSFV vaccines are usually administered to piglets around weaning age and to sows that later provide protections to piglets through maternal-derived antibodies (MDA) in colostrum^24^. Hence, a CSFV/PCV2 bivalent vaccine could be very useful and beneficial to the swine industry worldwide for the control of CSFV and PCV2 infections. In light of this, we developed a novel bivalent subunit vaccine based on full-length E2 and ORF2 proteins, which are the antigenic targets of CSFV and PCV2, respectively. The objective of this study was to evaluate the safety and efficacy of the CSFV/PCV2 bivalent vaccine in mice and specific pathogen-free (SPF) pigs.

## Results

### Verification of recombinant proteins and VLPs

**Figure 1:**
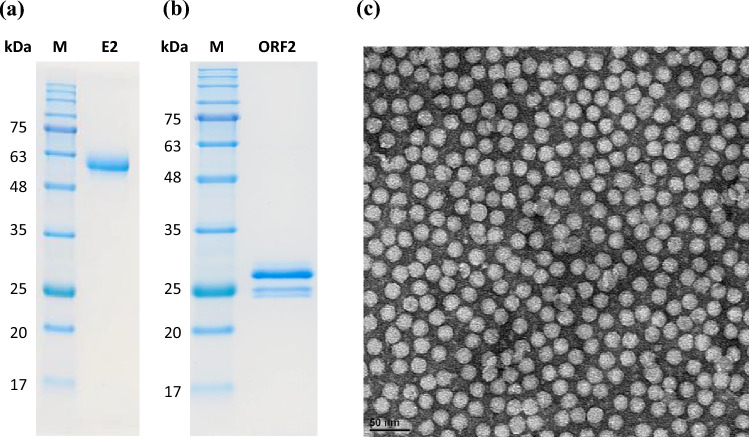
The characterization of expressed recombinant (**a**) CSFV E2 (52.9 kDa) and (**b**) PCV2 ORF2 (27.8 kDa) proteins separated in 12% SDS-PAGE gels. (**c**) The assembled PCV2 particles were negatively stained and observed by transmission electron microscopy. Scale bar: 50 nm (38,000 ×).

Two vaccine antigens, CSFV E2 and PCV2 ORF2, were used in this study. The former was expressed in Chinese hamster ovary cells (CHO cells), and the latter by baculovirus system. Purification of CSFV E2 and PCV2 ORF2 were verified by sodium dodecyl sulfate–polyacrylamide gel electrophoresis (SDS-PAGE) (Fig. 1a,b). The PCV2 ORF2 is capable of self-assembly into a virus-like particle (VLP) in vitro. The assembled PCV2 particles were visualized by transmission electron microscopy (Fig. 1c).

### The effect of bivalent vaccine on BALB/c mice in response to PCV2 challenge

To benchmark the CSFV/PCV2 bivalent vaccine against the CSFV E2 or PCV2 ORF2 monovalent vaccine, we first compared the immune responses induced by CSFV E2 alone, PCV2 ORF2 alone, and the CSFV E2 plus PCV2 ORF2 bivalent vaccine at various time points post-vaccination in BALB/c mice (Fig. S1a). CSFV-specific antibodies were evaluated at weeks 4 and 6. The results showed no significant differences in the CSFV-specific antibodies between the groups of CSFV E2 and the CSFV E2 plus PCV2 ORF2 bivalent vaccine (Fig. S1b). Compared to the PCV2 ORF2 monovalent vaccine, the titers of anti-PCV2 antibody and the neutralization antibody against PCV2 induced by the CSFV E2 plus PCV2 ORF2 bivalent vaccine were not significantly different (Fig. S1c). Four days after the PCV2 challenge, we did not observe a significant difference in PCV2 viremia between the CSFV E2 plus PCV2 ORF2 bivalent vaccine and the PCV2 ORF2 monovalent vaccine (Fig. S1d). Furthermore, the results of PCV2 ORF2 and CSFV E2 plus PCV2 ORF2 vaccine groups were comparable (Fig. S1c,d), indicating that CSFV E2 did not interact with PCV2 ORF2 adversely in the bivalent vaccine formula. In addition, mice immunized with CSFV vaccines showed a high level of PCV2 viremia after the virus challenge, suggesting that CSFV vaccines did not offer cross-protection against the PCV2 challenge.

### Safety of bivalent vaccine in pigs

Rectal temperatures and body weight gain (BWG) were measured at various time points after vaccination. Before the 1^st^ vaccination, the mean rectal temperatures in vaccinated and non-vaccinated groups were 40.1 ± 0.1 °C and 40.0 ± 0.2 °C, respectively. Compared to the non-vaccinated group, the rectal temperatures in the vaccinated group did not increase significantly, and no fever was observed within 48 h after the 1^st^ and 2^nd^ vaccinations (Fig. S2a,b). Twelve hours after the 2^nd^ vaccination, reddish lesions which were probably due to mild injection trauma were observed on the injection sites of vaccinated pigs but vanished 2 to 3 days later. In addition, there was no significant difference in mean BWG between vaccinated and non-vaccinated groups (Fig. S2c).

### Clinical characteristics of pigs after CSFV or PCV2 challenge

The protective immunity of the CSFV/PCV2 bivalent vaccine was evaluated by CSFV or PCV2 challenge in vaccinated and non-vaccinated pigs. Within 14 days post-CSFV challenge, the mean rectal temperatures of vaccinated pigs (VC group) were below 40℃, whereas non-vaccinated pigs (NC group) showed temperatures above 40.5 ℃ on days 4, 7 and 10, followed by a drop to 39.8℃ on day 14 due to progressive cachexia (Fig. S3a). Similar trends were shown in pigs challenged with PCV2, despite that no fever was observed within 7 weeks after the challenge (Fig. S3b). There was a significant difference in mean BWG between the VC and the NC groups at week 7, with the NC group showing negative figures (Fig. S3c). Such difference in mean BWG was not observed at week 12 in pigs challenged with PCV2 (Fig. S3d).

**Figure 2:**
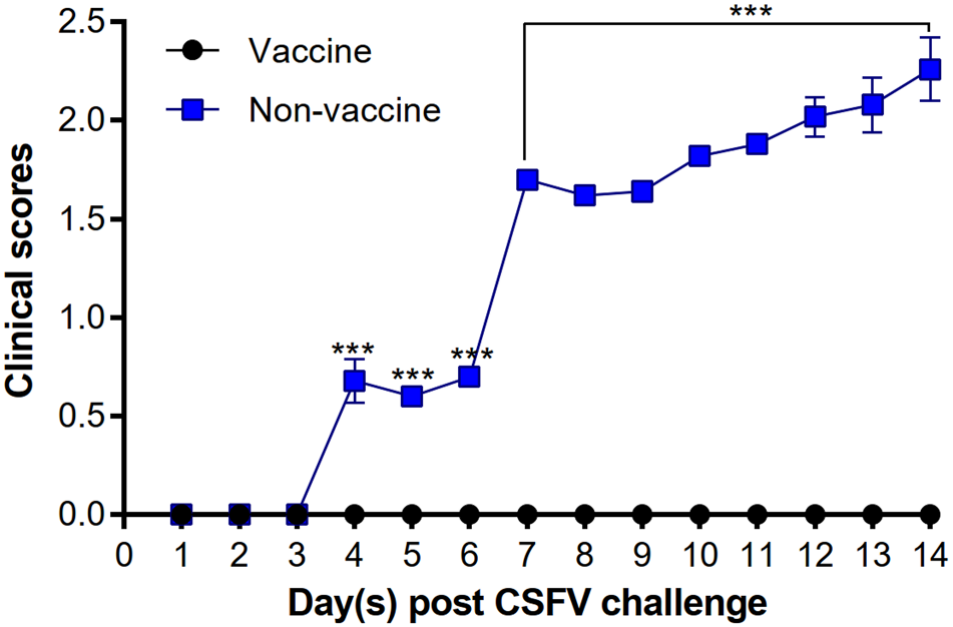
The clinical scores of pigs challenged with CSFV. The data are presented as mean ± SEM (n = 5 per group). Statistical significance between Vaccine and Non-vaccine groups: ^***^p < 0.001.

In the CSFV challenge experiment, typical clinical signs of CSFV infection appeared in non-vaccinated pigs on day 4 post-challenge and continued to deteriorate. Those pigs showed obvious depression, chills, unwillingness to move, and exhibited neurological signs on day 7 post-challenge. In contrast to vaccinated pigs showing no specific clinical signs, clinical scores of non-vaccinated pigs increased over time from days 4 to 14 after the CSFV challenge, and the difference between the two groups was statistically significant (Fig. 2). The pathological examinations of non-vaccinated pigs revealed moderate to severe non-suppurative meningoencephalitis, interstitial pneumonia, vasculitis in the liver, interstitial nephritis, marginal hemorrhages and infarctions in the spleen, and lymphoid depletion (Fig. S4). The lesion scores of the brain, tonsil, lung, spleen, various lymph nodes and ileocecal valve are presented in Table 1.

**Table 1:** Lesion scores of tissues from vaccinated and non-vaccinated SPF pigs in CSFV/PCV2 challenge studies.

	VC	NC	VP	NP
Brain	0.00 ± 0.00	2.20 ± 0.20^+++^	0.13 ± 0.10	0.00 ± 0.00
Tonsil	0.00 ± 0.00	2.00 ± 0.00^+++^	0.83 ± 0.06	0.77 ± 0.07
Lung	0.00 ± 0.00	1.60 ± 0.24^+++^	1.07 ± 0.24	2.47 ± 0.40***
Spleen	0.00 ± 0.00	1.40 ± 0.24^+++^	0.70 ± 0.17	1.43 ± 0.24***
HLN	0.00 ± 0.00	2.20 ± 0.20^+++^	0.53 ± 0.16	1.43 ± 0.24***
MLN	0.00 ± 0.00	1.80 ± 0.37^+++^	0.43 ± 0.08	1.10 ± 0.04***
ILN	0.00 ± 0.00	2.40 ± 0.51^+++^	0.73 ± 0.12	1.33 ± 0.05***
IV	0.00 ± 0.00	1.80 ± 0.37^+++^	0.23 ± 0.08	0.47 ± 0.12**

Lesions were scored from 0 (normal) to 4 (most severe) and presented as mean ± SEM (n = 5 per group). The vaccine groups were compared with their non-vaccinated counterparts, and statistical significance are presented as follows: ^+++^*p* < 0.001 (CSFV challenge study); **p < 0.01 and ***p < 0.001 (PCV2 challenge study). Abbreviations: HLN, hilar (trachea-bronchial) lymph nodes; ILN, inguinal lymph nodes; IV, ileocecal valve; MLN, mesenteric lymph nodes; VC & NC, vaccinated and non-vaccinated pigs challenged with CSFV; VP & NP, vaccinated and non-vaccinated pigs challenged with PCV2.

In the PCV2 challenge experiment, there were no specific clinical signs observed in the vaccinated pigs; non-vaccinated pigs gradually manifested signs of mild depression after virus infection with normal rectal temperatures. The main histopathological changes in PCV2-infected pigs were interstitial pneumonia in the lung and lymphoid depletion in various lymphoid tissues. The lesion scores of lung, spleen, and various lymph nodes in vaccinated pigs were significantly lower than those of corresponding organs of non-vaccinated pigs (Table 1).

### Quantitative RT-PCR analysis of serum and tissue samples

**Figure 3:**
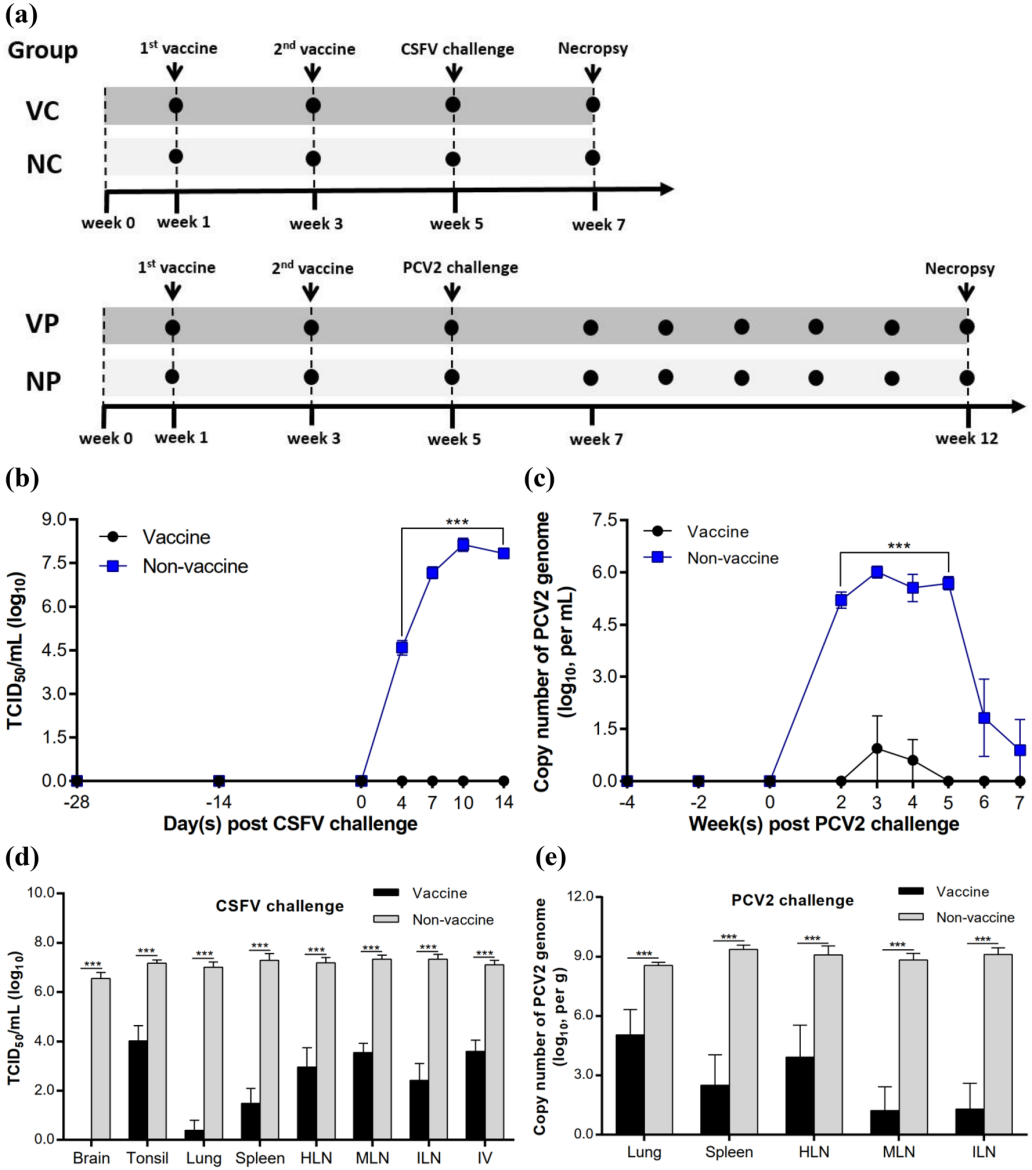
Protectivity of the CSFV/PCV2 bivalent vaccine against virus replication. (**a**) Schema of the experimental design of pig studies. VC and NC groups were challenged with CSFV while VP and NP groups were challenged with PCV2 four weeks after 1st vaccination. Black circle indicated the time of blood collection. Viral loads in serum (**b** and **c**) and various organ (**d** and **e**) samples were evaluated by quantitative PCR. The data are presented as mean ± SEM (n = 5 per group). Statistical significance between Vaccine and Non-vaccine groups: ^***^p < 0.001. Abbreviations: HLN, hilar (trachea-bronchial) lymph nodes; ILN, inguinal lymph nodes; IV, ileocecal valve; MLN, mesenteric lymph nodes; VC & NC, vaccinated and non-vaccinated pigs challenged with CSFV; VP & NP, vaccinated and non-vaccinated pigs challenged with PCV2.

The efficacy of the CSFV/PCV2 bivalent vaccine against viremia and virus replication was evaluated using qRT-PCR or qPCR. In the CSFV challenge study (Fig. 3a), no viremia was detected in the sera of vaccinated pigs, whereas non-vaccinated pigs developed high levels of viremia between day 4 and day 14 post-challenge (Fig. 3b). In the PCV2 challenge study (Fig. 3a), non-vaccinated pigs exhibited a high level of viremia 2 weeks after the challenge, which persisted for 3 weeks (Fig. 3c). In contrast, only a very low level of viremia was detected in vaccinated pigs at week 3 and week 4 post-challenge.

In both challenge studies, viral load was detected in the lung, spleen, and various lymphoid tissues (Fig. 3d,e). Furthermore, CSFV was also detected in the brains of non-vaccinated pigs (Fig. 3d). The viral load in tissues was significantly higher in non-vaccinated pigs. No CSFV load in sera and tissues was noted in PCV2-challenged pigs, and vice versa.

### Serum antibody assay

**Figure 4:**
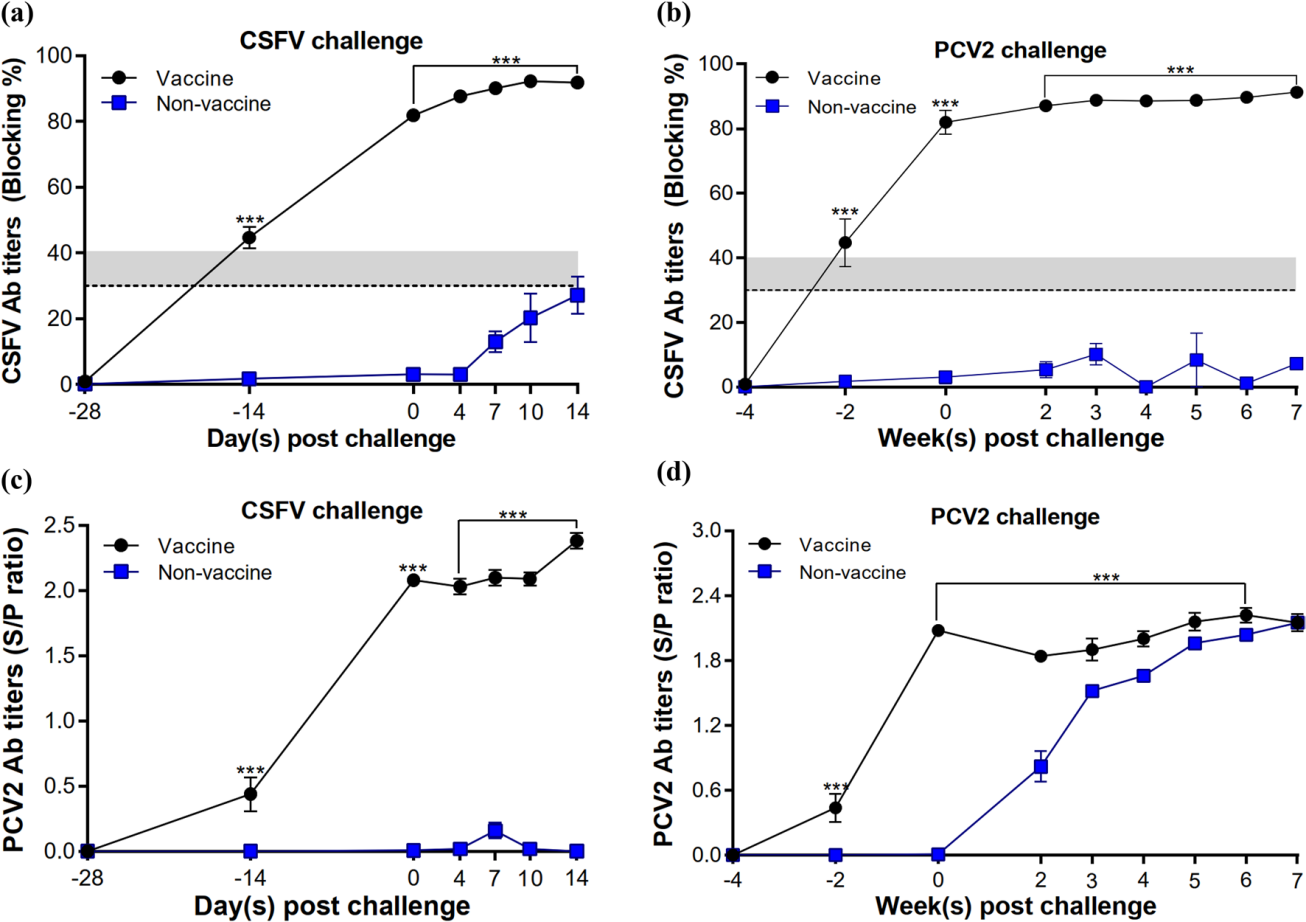
Antibody responses in pigs. CSFV-specific antibodies in pig sera were measured before and after (**a**) CSFV or (**b**) PCV2 challenges. PCV2-specific antibody responses before and after the (**c**) CSFV or (**d**) PCV2 challenges were also evaluated. All serum samples were analyzed separately. The data are presented as mean ± SEM (n = 5 per group). Statistical significance between Vaccine and Non-vaccine groups: ^***^p < 0.001.

**Figure 5:**
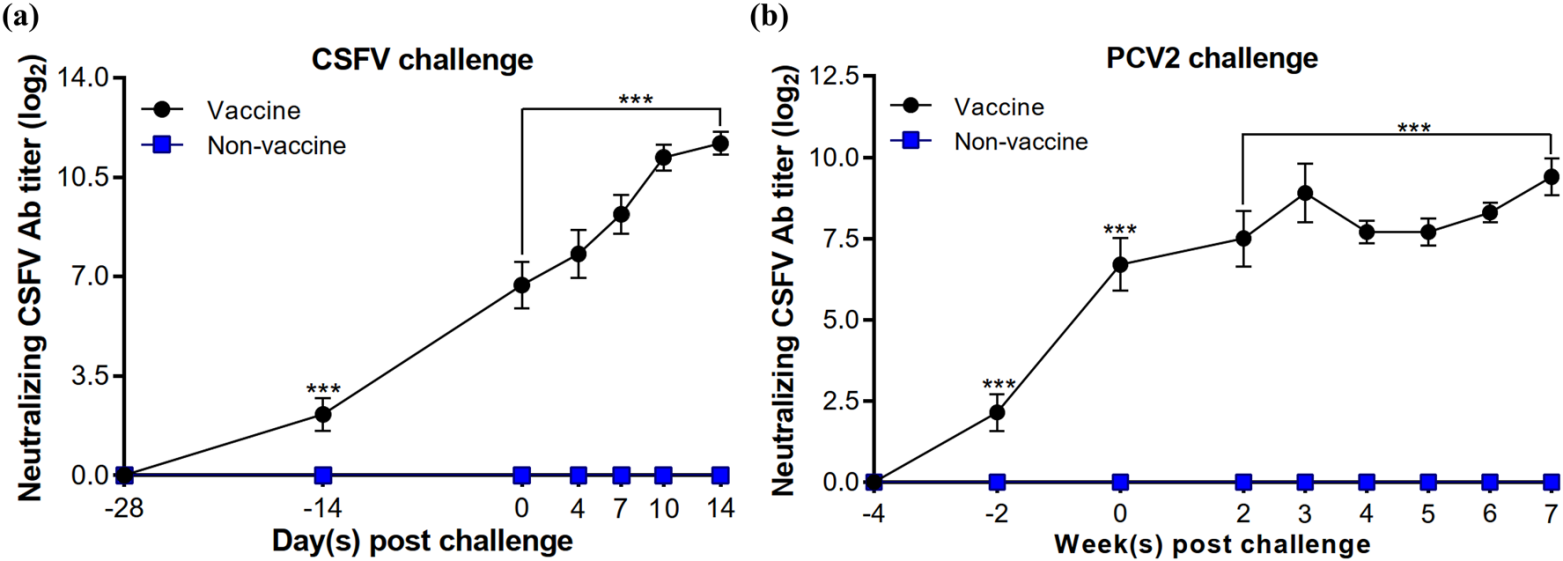
Neutralizing antibodies against CSFV in pig sera. All serum samples were separately analyzed before and after the (**a**) CSFV or (**b**) PCV2 challenges. The data are presented as mean ± SEM (n = 5 per group). Statistical significance between Vaccine and Non-vaccine groups: ^***^p < 0.001.

Pigs vaccinated with CSFV/PCV2 bivalent vaccine developed specific CSFV antibody responses, with the highest mean antibody blocking rates of 91.88 ± 1.09% and 91.30 ± 1.13% in CSFV and PCV2 challenged groups, respectively (Fig. 4a,b). In non-vaccinated pigs, CSFV antibody titer increased after the CSFV challenge, but not after PCV2 infection. The mean CSFV neutralization antibody titers in vaccinated pigs continued to increase after the challenge with CSFV or PCV2 (Fig. 5a,b). Notably, a strong anamnestic response was shown in vaccinated pigs challenged with CSFV (Fig. 5a). Infection of PCV2, however, attenuated such anamnestic response (Fig. 5b). The CSFV-specific antibody titers detected in vaccinated pigs were significantly higher than that of the non-vaccinated group at all time points.

Specific PCV2 antibodies were detected two weeks after the 1^st^ vaccination with a seroconversion rate of 20%, and all vaccinated pigs were positive for PCV2 antibodies at week 4 post-first vaccination. In pigs challenged with CSFV, the mean PCV2 antibody titers of vaccinated pigs were significantly higher than that of non-vaccinated pigs, with negative results shown in the latter group at nearly all time points (Fig. 4c). In pigs challenged with PCV2, PCV2 antibody titers increased in non-vaccinated pigs after the challenge and reached the same level as vaccinated pigs at week 7 post-PCV2 challenge when they were sacrificed. Until 6 weeks after the PCV2 challenge, significantly higher titers of PCV2 antibodies were noted in the vaccinated group (Fig. 4d).

## Discussion

Simultaneous administration of vaccines against four major pathogens of swine, including PCV2, has been demonstrated to be as efficacious as the administration of single vaccines^25^. To the best of our knowledge, no commercial CSFV and PCV2 bivalent vaccine is available. Although other experimental bivalent vaccines against CSFV and PCV2 which were developed using different approaches have been reported, only immunogenicity data were shown^26,27^, indicating that they were still in the early stages of development. In the present study, we demonstrated the immunogenicity and protection of a novel CSFV/PCV2 bivalent subunit vaccine by challenging vaccinated and non-vaccinated pigs with either CSFV or PCV2. This is in line with a recent study showing that a CSFV-PCV2 bivalent subunit vaccine elicited protective immune responses against CSFV and PCV2 challenges in pigs^28^. The promising results reported herein will encourage further development such as industrial production, field trials, and commercialization of this vaccine.

In terms of the ability of a vaccine to elicit antibodies that are sufficient to confer protective immunity, antigen selection is crucial in the process of vaccine production. In 2022, Chen et al. developed a bivalent vaccine through polycistronic baculovirus vector, showing the immunogenicity against CSFV-E2 and PCV2-Cap antigens in BALB/c mice^27^. However, it is unclear how efficient this bivalent vaccine is without a viral challenge experiment. In our PCV2 challenge test in BALB/c mice, the results demonstrated that the novel CSFV/PCV2 bivalent subunit vaccine significantly reduced PCV2 viremia on both days 2 and 4 post-challenge.

Mammalian cells, especially CHO cell line, have been considered as the optimal host cells for the production of antibodies, cytokines, and viral antigens. Recently, Feng et al. developed stable transgenic CHO cells to express recombinant CSFV E2 protein^29^. In this study, the E2 antigen was expressed by stable clones derived from CHO cells followed by a series of purification processes to yield high-quality pure E2 proteins. The PCV2 component of the bivalent vaccine was produced using the baculovirus expression system. Baculovirus-based protein expression in insect cells has been recognized to be an effective tool for VLP production^30^. Due to their size and shape which mimic those of native viruses, VLPs can efficiently elicit immune responses^31^. The baculovirus expression system developed in this study successfully generated PCV2 VLPs. The immunogenicity and protection of PCV2 VLPs were evaluated by animal studies which showed satisfactory results.

In the CSFV study, the seroconversion rate of neutralizing antibodies against CSFV reached 80% two weeks after the vaccination of pigs with the CSFV/PCV bivalent vaccine. Four weeks after vaccination, all vaccinated pigs showed seroconversion with a high mean neutralizing antibody titer of Log_2_ 6.7. During the 14-day observation period, no typical CSFV clinical signs and viremia were detected in vaccinated pigs. There were also no typical CSF lesions observed in vaccinated pigs 14 days after CSFV challenge. Significant reductions (in the range of 3.2 to 6.6 Log_10_ TCID_50_/mL) of CSFV loads in various organs were observed in vaccinated pigs as compared to non-vaccinated pigs. Death of non-vaccinated pigs was not observed within 14 days after the virulent CSFV challenge. According to the literature, pigs infected with CSFV died as late as five weeks after acute-lethal courses^32^. In Choe’s study, five pigs in the mock group died between 12 and 19 days post CSFV inoculation (dpi), but only one died before 14 dpi^33^. Hence, it is speculated that non-vaccinated pigs in the NC group would die after the end of the study along with the increase of time post CSFV challenge.

The high efficacy of the CSFV/PCV2 bivalent vaccine in protecting pigs from PCV2 infection was also demonstrated in the current study. The seroconversion rate of neutralizing antibodies against PCV2 reached 100% with a mean PCV2 ELISA titer of 2.1 (S/P ratio) four weeks after the vaccination of pigs. No clinical signs were observed in vaccinated pigs after the PCV2 challenge. Significant reductions in viremia levels, lesion scores and viral loads in the lung, spleen and various lymph nodes were also observed in vaccinated pigs as compared to those of non-vaccinated pigs. The results described above demonstrate that the CSFV/PCV2 bivalent vaccine is efficient in protecting pigs against CSFV/PCV2 challenge after two vaccinations with an interval of 2 weeks.

In this study, we have demonstrated that administration of the CSFV/PCV2 bivalent vaccine offered protection in pigs that were later challenged with CSFV or PCV2. The efficacy of the bivalent vaccine against CSFV and PCV2 challenge was not interfered with by the pre-existing PCV2-derived and CSFV-derived immune responses, respectively. The new CSFV/PCV2 bivalent vaccine offers potential for increasing vaccination coverage in pigs.

Our results showed that two doses of the bivalent vaccine protected pigs from CSFV or PCV2 challenge, as shown by antibody immune response and reduction in clinical scores and viremia when compared to the non-vaccinated group. One bivalent vaccine dose has been proven to provide protection against PCV2 challenge in our mouse experiment. We will further evaluate the efficacy of a single dose of the CSFV/PCV2 bivalent vaccine. Moreover, a further comparative study using commercial vaccines may also provide a valuable insight into the application of this novel bivalent vaccine.

In conclusion. the CSFV/PCV2 bivalent vaccine is safe and effective against CSFV or PCV2 challenge. The findings of this study may provide valuable information on the potential use of the CSFV/PCV2 bivalent vaccine in contribution to the eradication plan of CSFV/PCV2 infections.

## Methods

### Preparation of CSFV E2 and PCV2 ORF2 recombinant proteins and vaccines

The CSFV and PCV2 vaccine antigen design was conducted based on their protein sequences with NCBI accession numbers of AAT66638 and ABV21950, respectively. Briefly, the CSFV E2 was cloned and expressed in CHO cells. The E2 recombinant protein antigens were then purified by nickel-chelating affinity chromatography. Recombinant baculovirus carrying the ORF2 gene of PCV2 (PCV2 ORF2) was used to infect High Five™ cells to express PCV2 ORF2 protein, which were purified by ion exchange chromatography. SDS-PAGE and transmission electron microscopy were used to verify protein production and VLP formation, respectively. The monovalent and bivalent vaccines were prepared by mixing antigen with an oil adjuvant, Montanide™ ISA-206 (Seppic, France), in a 1:1 ratio w/w (antigen: adjuvant).

### Ethical statement

All animal experiments in this study were performed in accordance with the relevant guidelines and regulations. Studies with live CSFV were conducted in biosafety level 3 facilities, and experimental animals were kept in high containment animal biosafety level 3 facilities. The animals were sacrificed following the AVMA guidelines for the euthanasia of animals (Version 2020.0.1). The studies involving mice and pigs were reviewed and approved by the Institutional Animal Care and Use Committees (IACUC) of the National Ilan University (Committee protocol number 111–4) and the National Pingtung University of Science and Technology (NPUST-IACUC NPUST-111–01), respectively. All procedures in the current study were reported in compliance with the ARRIVE guidelines 2.0 (https://arriveguidelines.org/).

### Mouse immunization with the vaccine candidate

Five-week-old BALB/c mice were purchased from BioLASCO Taiwan Co., Ltd. After one week of a regular diet, the mice were randomly divided into four groups (n = 5 each group: (1) PBS control group; (2) CSFV E2 monovalent vaccine group; (3) PCV2 ORF2 monovalent vaccine group; and (4) CSFV E2 plus PCV2 ORF2 bivalent vaccine group). For immunization, the mice were intramuscularly injected with 0.1 mL of CSFV E2 (15 μg/mL), PCV2 ORF2 (50 μg/mL), or CSFV E2 (15 μg/mL) plus PCV2 ORF2 (50 μg/mL), whereas mice in the PBS control group was given the same volume of treatment vaccines. The dose was determined based on our previous PCV2 studies (unpublished) and publicly available information of the antigen contents of other CSFV E2 vaccines. Three weeks post-vaccination, the vaccinated mice were challenged with 10^6.3^ TCID_50_ PCV2 (Fig. S1a). At the end of experiment, the mice were sacrificed using carbon dioxide inhalation, and their blood was collected.

### Measurement of specific antibodies and viremia in mice

The blood samples were collected through the submandibular vein at weeks 4 and 6, including both day 2 and day 4 post PCV2 challenge. After centrifugation at 4 °C, the mouse sera were isolated and stored at -80 °C for future experiments.

Anti-CSFV specific antibodies were evaluated using a commercial ELISA kit, Classical Swine Fever Antibody Test Kit (SK106 CSFV E2, BioChek, The Netherlands). Measurement of anti-PCV2 specific antibodies were conducted by immunofluorescence assay (IFA)^34^.

To perform the neutralization test, the mouse serum samples were incubated with PCV2 along with porcine kidney 15 (PK15) cells for 3 days. After fixation with 80% acetone, PK15 cells were incubated with serum obtained from a New Zealand white rabbit immunized with purified and inactivated PCV2, followed by FITC labeled anti-rabbit antibodies (Sigma-Aldrich). The neutralizing antibody titer for PCV2 was calculated using the 50% virus neutralization test (VNT50)^35^.

The PCV2 viremia was detected by extraction of PCV2 nucleic acids in mouse sera with LabPrep™ Viral DNA Mini Kit. The nucleic acid samples were then analyzed using SBC Porcine Circovirus Type 2 qPCR Kit (Schweitzer Biotech Company Ltd., Taiwan).

### Vaccination and challenge of pigs

Twenty cesarean-derived, colostrum-deprived, 4-week-old SPF pigs purchased from Agricultural Technology Research Institute (ATRI), Taiwan were randomly assigned into 4 groups (n = 5 each group: (1) Vaccine + CSFV (VC) group; (2) Non-vaccine + CSFV (NC) control group; (3) Vaccine + PCV2 (VP) group; (4) Non-vaccine + PCV2 (NP) control group). After one-week observation, each pig in the treatment groups was intramuscularly immunized with 2 mL of the CSFV/PCV2 bivalent vaccine, containing 50 μg of CSFV E2 and 50 μg of PCV2 ORF2 antigens, twice with an interval of 2 weeks, whereas pigs in the control groups were given the same volume of normal saline (0.9% NaCl) (Fig. 3a). To monitor the safety of the CSFV/PCV2 bivalent vaccine in pigs, the rectal temperatures, BWG, clinical signs, and CSFV/PCV2 nucleic acids of each pig were measured before sacrifice at week 7 (CSFV challenge) or week 12 (PCV2 challenge). The monitoring periods after viral challenges were not the same because the disease progression of the two viruses were different. A mean rectal temperature over 40.5℃ was considered as fever^36^.

One week before challenge, pigs in VC and NC groups were transferred to Animal Health Research Institute for CSFV challenge; VP and NP groups were transferred to National Pingtung University of Science and Technology in Taiwan for PCV2 challenge. Previously established challenge procedures were used. For CSFV challenge, four weeks after the 1^st^ vaccination, pigs in VC and NC groups were intramuscularly challenged with 2 mL of CSFV ALD strain (1 × 10^5.41^ FAID_50_). For PCV2 challenge, pigs in VP and NP groups were administered with 2 mL of PCV2d THF0601-7 strain (1 × 10^6^ TCID_50_ /mL) via intranasal and intramuscular routes on the first day of challenge, followed by intranasal inoculation of 1 mL of viral suspension using a syringe on 2 consecutive days. The pigs were euthanized through exsanguination under a surgical plane of anesthesia induced by intramuscular administration of Stresnil® (Azaperone), followed by intravenous Zoletil® (tiletamine-zolazepam mixture) injection. Tissues were harvested and fixed for future examination.

### Pathological and clinical examination

For histopathological examination, tissue sections were fixed in 10% neutral-buffered formalin. The scoring system of 0 (normal) to 4 (most severe) was used for the evaluation of the severity of lymphoid depletion, interstitial pneumonia, infarcts and hemorrhages in the spleen. In addition, clinical signs of CSFV were evaluated by a scoring system of 0 (normal) to 3 (most severe)^37^. The histopathological changes and clinical syndromes mentioned above were estimated by two veterinary pathologists blinded to treatment allocation.

### Serological examination

All pig serum samples were isolated and measured for the presence of anti-CSFV or anti-PCV2 antibodies. For the detection of CSFV-specific antibodies, a commercial ELISA kit, IDEXX CSFV Ab Test Kit (IDEXX Laboratories, Inc., USA), was used to perform the tests. The CSFV ELISA antibody levels were expressed as the blocking % which ≦ 30 and ≧ 40 were interpreted as negative and positive, respectively. A blocking % between 30 and 40 was considered as suspected. The neutralization antibody titer against CSFV ALD strain was determined in duplicate wells and expressed as the log_2_ of the reciprocal of the highest serum dilution that 50% of the wells were protected from infection, calculated using the Reed-Muench Method. A Porcine Circovirus type 2 Antibody Test Kit (SK105, BioChek), which is an ELISA kit, was used to detect PCV2-specific antibodies according to the manufacturer’s recommended procedures.

### Quantitative RT-PCR for viral quantification

CSFV RNA was extracted from serum and tissue samples using MagNA Pure 24 Total NA Isolation Kit (Roche Molecular Systems, Inc., USA). The quantitative reverse transcription PCR (qRT-PCR) was performed with the LightCycler Multiplex RNA Virus Master Kit (Roche Diagnostics, Mannheim, Germany) according to the manufacturer’s instructions. A CSFV-containing blood sample of known concentration was ten-fold serially diluted from 10^8^ to 10^1^ TCID_50_/mL and used to establish a standard curve.

PCV2 DNA was extracted using AxyPrep Body Fluid Viral DNA/RNA Miniprep Kit (Axygen®; Corning, USA). The qPCR was performed using PowerUp SYBR Green Master Mix (Applied Biosystems™, Thermo Fisher Scientific Inc., USA) on QuantStudio™ 3 Real-Time PCR Systems (Applied Biosystems™, Thermo Fisher Scientific Inc., USA). Viral DNA concentrations were expressed as Log_10_ PCV2 genomic copies/mL in serum and tissue.

The specific primer and/or probe sets used for the detection of CSFV and PCV2 are summarized in Table S1.

### Statistical analysis

Data analyses were performed using the generalized linear models (GLM) procedure of SAS® (version 9.4, SAS Institute Inc., Cary, NC, USA). A two-sided *p* value of < 0.05 was considered statistically significant.

### Supplementary Information

Supplementary Information.

## Data Availability

The datasets generated during and/or analyzed during the current study are available from the corresponding author on reasonable request.
