# Supplementary material for: Efficacy evaluation of a bivalent subunit vaccine against classical swine fever virus and porcine circovirus type 2
Source: Sci Rep. 2024 Feb 6;14:2997. doi: 10.1038/s41598-024-53624-w (PMC10844208; doi:10.1038/s41598-024-53624-w)
Supplement: Supplementary file 1 — Supplementary Information. [file 41598_2024_53624_MOESM1_ESM.pdf]

Efficacy Evaluation of a Bivalent Subunit Vaccine Against Classical Swine Fever Virus and Porcine Circovirus Type 2

Yu-San Chen, Chang-Ye Lee, Chi-Chien Wu, Pei-Lun Kao, Tai-An Chen, Yahui Huang, Wen-Bin Chung, Tsun-Yung Kuo, Charles Chen

Table of Contents

Supplemental Figures..2

Supplemental Table..9

SUPPLEMENTAL FIGURES

(a)

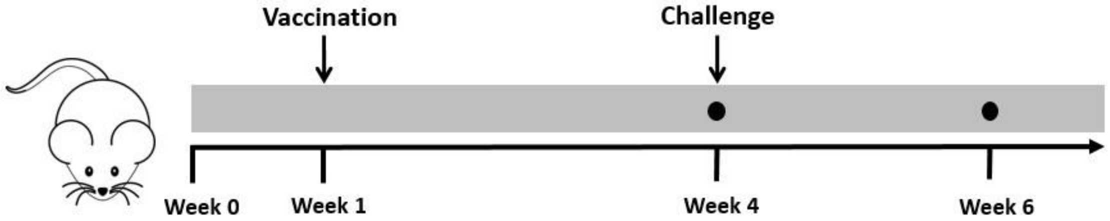

(b)

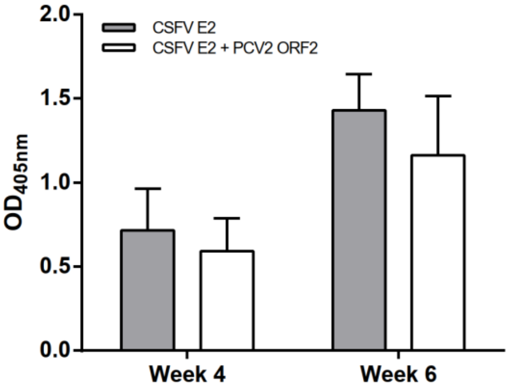

(c)

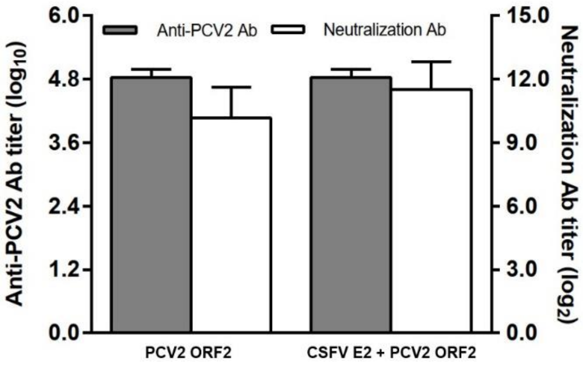

(d)

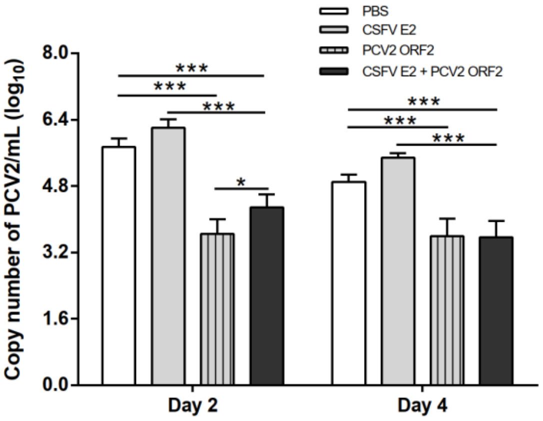

Figure S1. The effect of the CSFV/PCV2 bivalent vaccine on BALB/c mice. (a) Experimental design: the mice in treatment groups were vaccinated at week 1 and challenged with $10^{6.3}$ $TCID_{50}$ PCV2 at week 4. The black circle indicates the time of blood collection. All serum samples were separately analyzed. (b) Detection of CSFV-specific antibodies by ELISA at weeks 4 and 6. (c) Measurement of anti-PCV2

specific antibodies using immunofluorescence assay and evaluation of PCV2 neutralization antibody responses at week 4. (d) PCV2 viremia test on days 2 and 4 post-challenge. The data are presented as mean ± SEM (n = 4-5 per group). Statistical significance: $^{*}p < 0.05$, $^{***}p < 0.001$. Abbreviations: CSFV, classical swine fever virus; PCV2, porcine circovirus type 2.

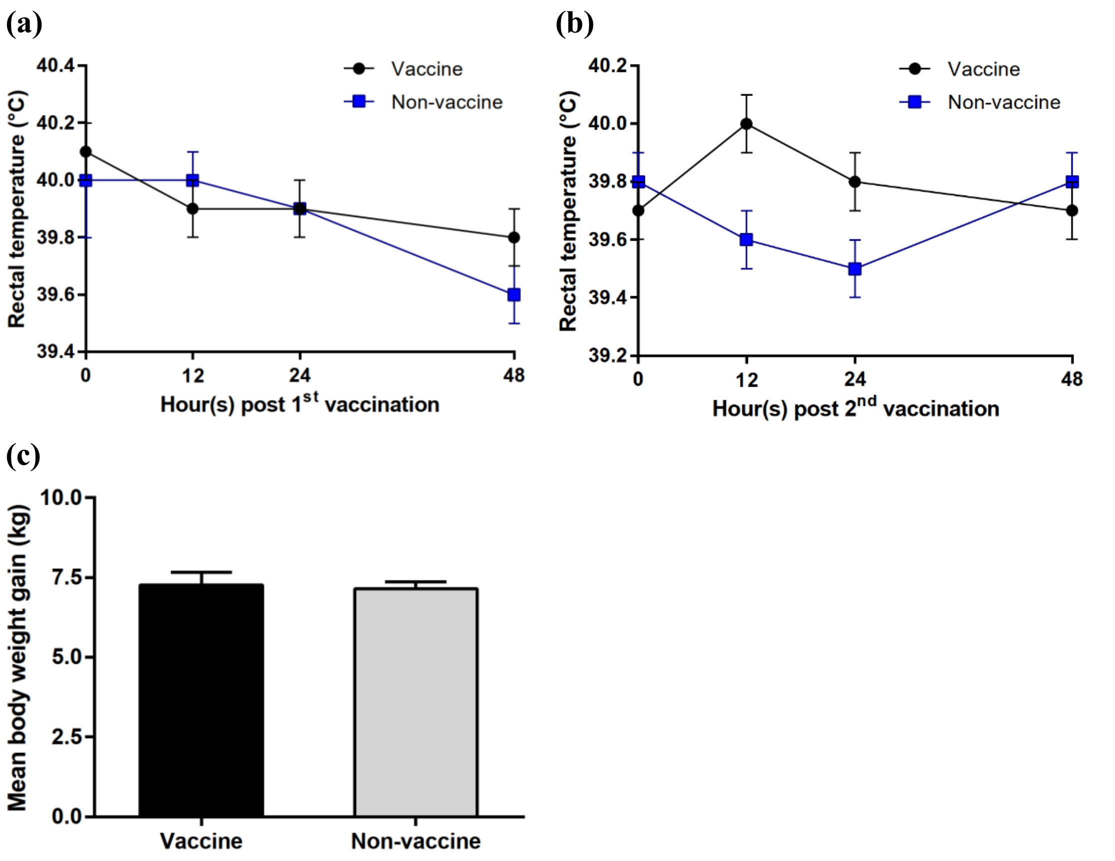

Figure S2. Safety assessment of the CSFV/PCV2 bivalent vaccine before virus challenge. The mean rectal temperatures in pigs were measured before and after (a) the 1st and (b) the 2nd vaccinations. (c) The mean body weight gain in vaccinated and non-vaccinated pigs were measured at week 5. The data are presented as mean ± SEM (n = 10 per group).

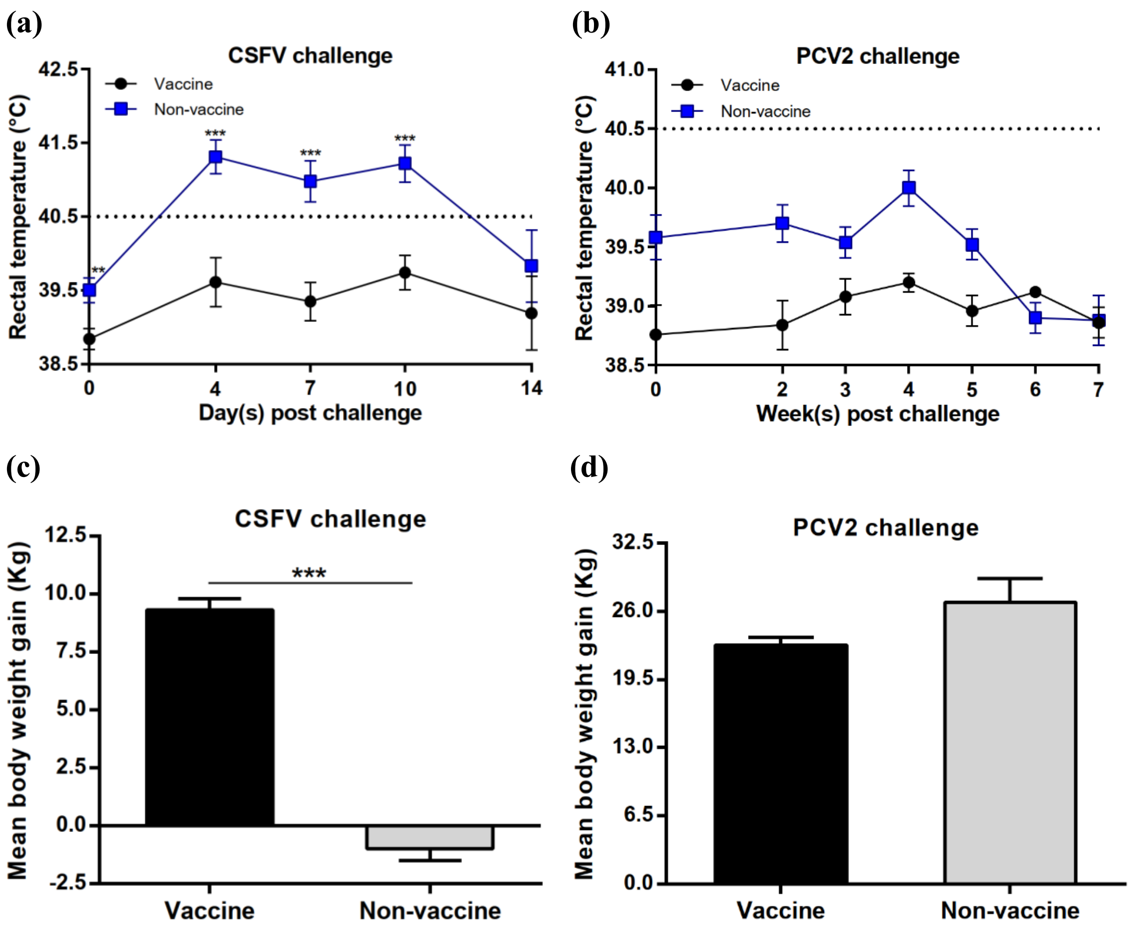

Figure S3. Clinical characteristics of the rectal temperatures and body weight gains for vaccinated and non-vaccinated SPF pigs challenged with CSFV (a and c) or PCV2 (b and d). The data are presented as mean ± SEM (n = 10 per group). Statistical significance: $^{**}p$ <0.01, $^{***}p$ <0.001. Abbreviations: CSFV, classical swine fever virus; PCV2, porcine circovirus type 2.

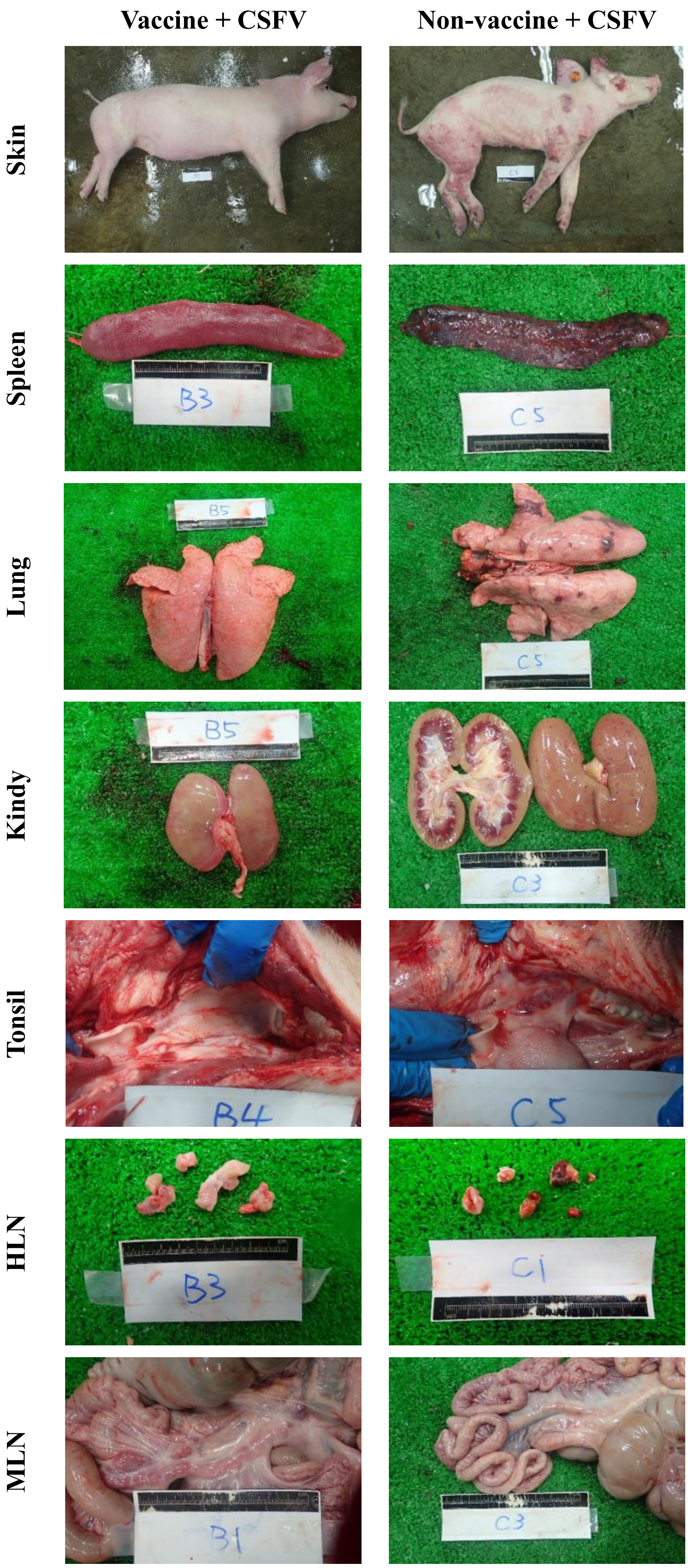
Vaccine + CSFV
Non-vaccine + CSFV
Skin
Spleen
Lung
Kindy
Tonsil
HLN
MLN

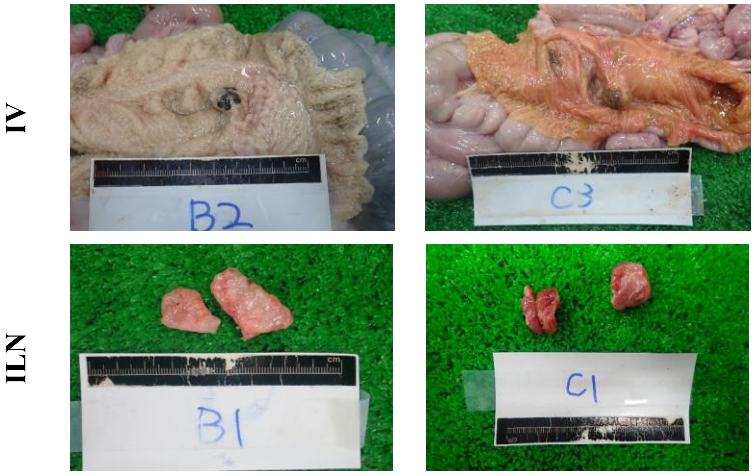

Figure S4. The macroscopic lesions of visceral organs at necropsy. After the CSFV challenge, the non-vaccine group presented with pathological lesions of CSFV infection (n = 5 per group). Abbreviations: CSFV, classical swine fever virus; HLN, hilar (trachea-bronchial) lymph nodes; ILN, inguinal lymph nodes; IV, ileocecal valve; MLN, mesenteric lymph nodes.

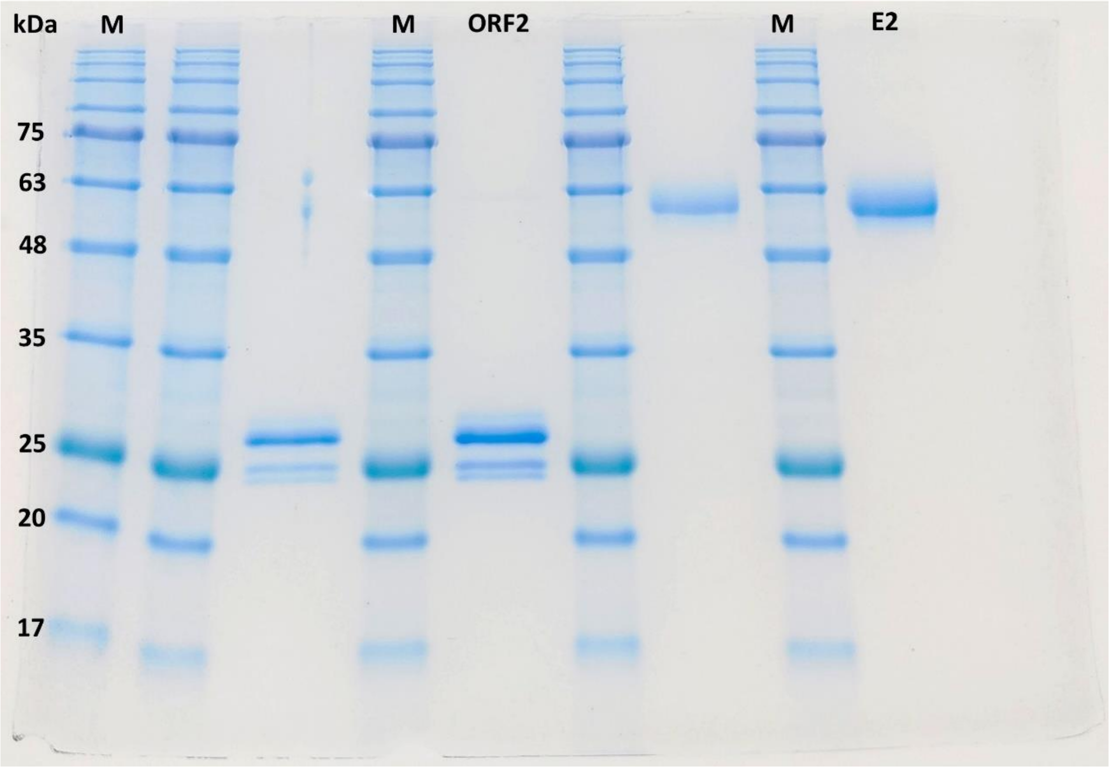

Figure S5. Full SDS-PAGE gel showing the expressed recombinant CSFV E2 (52.9 kDa) and PCV2 ORF2 (27.8 kDa) proteins that are presented in Figures 1a and 1b, respectively.

SUPPLEMENTAL TABLE

Table S1. Quantitative RT-PCR primer and probe sequences for detection of CSFV and PCV2 in serum and tissue samples.

Target		Sequences (5'-3')
CSFV	Forward	ATGCCCAYAGTAGGACTAGCA
	Reverse	CTACTGACGACTGTCCTGTAC
	Probe	FAM-TGGCGAGCTCCCTGGGTGGTCTAAGT-TAMRA
PCV2	Forward	ACATCGAGAAAGCGAAAGGA
	Reverse	ACGTTACAGGGTGCTGCTCT

Abbreviations: CSFV, classical swine fever virus; PCV2, porcine circovirus type 2.
